# Anatomical and Functional Factors Influencing Recovery in Idiopathic Epiretinal Membrane After Surgery

**DOI:** 10.3390/diagnostics16081204

**Published:** 2026-04-17

**Authors:** En-Jie Shih, Kai-Ling Peng, Ya-Hsin Kung, Tsung-Tien Wu

**Affiliations:** 1 Department of Ophthalmology, Kaohsiung Veterans General Hospital, Kaohsiung 813, Taiwan; 2 Department of Optometry, Shu-Zen Junior College of Medicine and Management, Kaohsiung 821, Taiwan; 3 School of Medicine, National Yang Ming Chiao Tung University, Taipei 112, Taiwan

**Keywords:** idiopathic epiretinal membrane, internal limiting membrane, central foveal thickness, vitrectomy, peeling

## Abstract

**Background**: Idiopathic epiretinal membranes (iERMs) are treated with pars plana vitrectomy and epiretinal membrane peeling. Simultaneous internal limiting membrane (ILM) peeling could reduce the recurrent rate. We aimed to explore the anatomical and functional factors influencing recovery outcomes in patients with iERM after surgical intervention. **Methods**: We enrolled 85 eyes of 85 participants with iERM who underwent pars plana vitrectomy with ERM and ILM peeling from January to December 2020 in Kaohsiung Veterans Hospital. We analyzed ERM staging, preoperative and postoperative vision and findings of retinal microstructures, and thickness changes determined using optic coherence tomography (OCT), as well as pre- and postoperative inner and outer retinal layer thickness changes. **Results**: The mean age was 65.64 ± 6.19 years, and no ERM recurrence was observed within one year. Males comprised 44.71% of participants (38/85). The mean preoperative vision score was 0.47 [Snellen equivalent (SE), 68/200] ± 0.29 logMAR, and the mean final vision score was 0.32 (SE, 96/200) ± 0.30 logMAR. Visual improvements were significant (*p* < 0.001, paired *t*-test). Preoperative vision (*β* = 0.327, *p* = 0.010) and final lens status (*β* = 0.400, *p* = 0.002) were significantly correlated with final vision (*R*^2^ = 0.309). Central foveal thickness and inner and outer retinal layer thickness decreased continuously until 12 months postoperatively in the pseudophakia group, whereas for those in the phakia group, the outer retinal layer thickness only decreased in the first 6 months. **Conclusions**: Poor initial vision and final phakia significantly worsened final visual outcomes. Postoperative vision, central foveal thickness, and thickness of the inner and outer retinal layers showed the continuous statistical improvement in pseudophakic eyes over 6 months.

## 1. Introduction

Epiretinal membrane (ERM) refers to the proliferation of nonvascular fibrocellular tissue covering the innermost retinal internal limiting membrane (ILM) [1], particularly in the macular area, where it is also known as macular pucker. ERM is classified according to etiology as (1) idiopathic ERM (iERM), which develops in the absence of pre-existing ocular pathologies and is frequently associated with posterior vitreous detachment (PVD), and (2) secondary ERM, which occurs due to coexisting ocular diseases, including diabetic retinopathy, retinal vein occlusion, and peripheral retinal breaks [2,3,4,5], ocular treatments, including photocoagulation and intravitreal injections; and intraocular surgeries, such as cataract and vitreoretinal surgeries. The prevalence of iERM is 7–11.8%, and it is increasing in elderly patients over 50 years of age [3,4].

Several ERM grading systems have been proposed based on different manifestations as follows: the Gass grading system based on fundus presentations, Mathews’ study of foveal thickness using optical coherence tomography (OCT) findings, and Govetto’s four-stage classification system of OCT morphologic retinal changes [6,7,8]. The gold standard treatment for ERM with different causes and severity is plana vitrectomy (PPV), followed by ERM peeling with or without ILM peeling, which results in relatively good outcomes, with the improvement of more than two lines of vision in 80% of patients [1].

The outcomes of visual function and retinal anatomy can vary with universal surgical approaches [9]. Several studies have attempted to evaluate symptoms, visual function, and OCT parameters such as central foveal thickness (CFT) and ellipsoid zone integrity as prognostic factors in patients with ERM [10,11,12,13]. In this study, we further evaluated the preoperative and postoperative changes in the thickness of the inner and outer retinal layers under different lens conditions. We aimed to determine the anatomical and functional variations and recovery following ERM and ILM removal in iERM.

## 2. Materials and Methods

### 2.1. Inclusion and Exclusion Criteria

This study included patients with ERM who underwent PPV, ERM, and ILM peeling between January 2020 and December 2020. The study was approved by the institutional review board of Kaohsiung Veterans General Hospital (approval number 24-CT4-01) and designed in accordance with the Declaration of Helsinki. Only one eye that underwent surgical intervention was included in the study to ensure the independence of the statistical observations. We reviewed the charts of all cases with histories and records of anterior and posterior segments. The exclusion criteria were as follows: (1) preoperative significant cataract (graded more severe than nuclear color 3 and nuclear opalescence 3) [14]; (2) combination surgery of vitrectomy and cataract surgeries; (3) high myopia (axial length > 26 mm or refractive error < −6 diopter); (4) glaucoma surgery or treatment, (5) ERM with lamellar hole; and (6) secondary ERM related to uveitis, retinal detachment, vascular disease, age-related macular degeneration, ocular trauma, and other non-cataract ocular surgeries.

### 2.2. General Data and Microstructural Assessment by OCT

**Figure 1:**
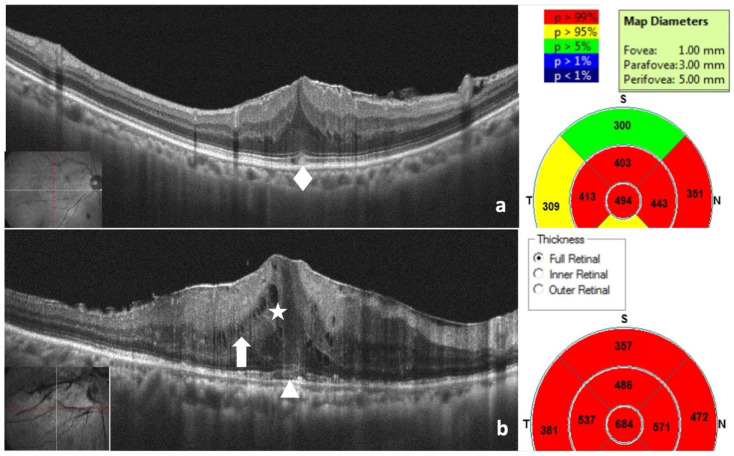
(**a**) This case represents a preoperative stage 3 ERM with no foveal pit and the presence of EIFL. In OCT images, a central bouquet of cotton ball signs, emphasized by a diamond, was observed at the outer retinal layer of the fovea. The mean CFT was measured within a 1.0 mm diameter area, encompassing the foveal pit, slope, and avascular zone. (**b**) This is a preoperative stage 4 case with the absence of a foveal pit, the presence of EIFL, and disrupted retinal layers. Intraretinal cysts, characterized by hypo-reflective and round-shaped intraretinal fluid, are marked with a star. Schisis-like changes, consisting of hypo-reflective longitudinal intraretinal fluid combined with tractional fibers, are shown with an arrow. The EZ and ELM are distorted and disrupted, indicated by a triangle. The IRLT and ORLT were measured using OCT based on various thickness options.

Best-corrected visual acuity (BCVA) was measured using Snellen charts and converted into the logarithm of the minimum angle of resolution (logMAR) for data analysis. All patients were evaluated using spectral-domain OCT (RTVue Scanner; Optovue, Inc., Fremont, CA, USA) for retinal microstructural characteristics such as intraretinal round and schisis-like cysts, central bouquet, integrity of the ellipsoid zone (EZ), and the whole retinal layer thickness including inner (I) and outer (O) retinal layer thickness (RLT). Postoperative follow-ups were performed at 1, 3, 6, and 12 months. ERM was graded using the four-stage ERM classification system [8], which accounts for the absence of a foveal pit, ectopic inner foveal layers (EIFLs), and retinal layers disorganization. The ERM stages are as follows: Stage 1: Normal foveal pit. Stage 2: Absent foveal pit. Stage 3: Absence of foveal pit and presence of EIFL (Figure 1a). Stage 4: Absence of foveal pit with the presence of EIFL and disrupted retinal layers (Figure 1b). The EIFL is defined as a continuous hyporeflective or hyperreflective band extending from the inner nuclear layer (INL) and inner plexiform layer (IPL) across the fovea [8].

In this study, we defined the retinal layer according to the OCT stratification. The inner retinal layer (IRL) extends from the innermost nerve fiber layer outward to the end of the inner nuclear layer. Conversely, the outer retinal layer (ORL) extends outward from the outer plexiform layer to the retinal pigment epithelium/Bruch complex (Figure 1b). We measured the CFT as the mean foveal thickness within a 1.0 mm diameter area, including the foveal pit, slope, and avascular zone.

Intraretinal cysts refer to hypo-reflective and round-shaped intraretinal fluid (Figure 1b). Schisis-like changes refer to hypo-reflective longitudinal intraretinal fluid combined with tractional fibers, presented on OCT images (Figure 1b). According to Govetto et al., a central bouquet is defined as a 100 μm diameter zone that manifests on OCT [15]. It has one of three appearances: (1) Cotton ball sign: A thickened, fuzzy hyperreflective area between the ellipsoid zone (EZ) and interdigitation zone (Figure 1a). (2) Acquired vitelliform lesion: Dome-shaped subretinal hyperreflective material between the EZ and retinal pigment epithelium. (3) Foveolar detachment.

### 2.3. Surgical Procedures

All patients underwent a standard 23- or 25-gauge pars plana vitrectomy. After core vitrectomy, the epiretinal membranes were peeled if present, followed by ILM peeling. As Brilliant Blue G was not available in Taiwan at that time, we used 0.05% Indocyanine Green, which was washed out less than 30 s, for ILM staining, and informed the patients of the possible toxicities. ILM peeling was performed in a circular area centered on the fovea, extending to the margin of the optic disk and remaining within the boundaries of the major vascular arcades.

### 2.4. Statistical Analysis

We used the Pearson correlation test to compare final vision and CFT with the continuous variables. Independent *t*-tests were conducted to compare the final vision and CFT with the categorical variables. Two and three groups were created according to the preoperative and postoperative lens statuses (pseudophakia versus phakia/phakia versus phakia/pseudophakia). In addition to univariate analyses, stepwise multiple linear regression was constructed to identify independent factors of functional (final vision) and anatomical (final CFT) outcomes. To address potential confounding, postoperative lens status and follow-up duration were included in the models to evaluate their independent contributions to the final results. All factors within univariate analysis were entered into the regression. Multicollinearity was strictly monitored using the Variance Inflation Factor (VIF), with a VIF < 10 considered acceptable for inclusion. Data were analyzed using SPSS statistical software (version 31.0; IBM, Armonk, NY, USA). *p* < 0.05 was considered significant.

## 3. Results

### 3.1. General Data Analysis

In this study, 85 eyes of 65 patients (mean age: 65.64 ± 6.19 years; males: 46.15%, 30/65) with iERM underwent PPV and both ERM and ILM peeling. Bilateral eye involvement accounted for 24.0% (20/85). Preoperative conditions included pseudophakia (42.35%, 36/85), ERM stage III and IV (78.82%, 67/85), intraretinal cysts (80%, 68/85), schisis-like cysts (18.82%, 16/85), EZ distortion (16.47%, 14/85) and central bouquet (41.18%, 35/85). The mean PV was 0.47 [Snellen equivalent (SE), 68/200] ± 0.29 logMAR, and the mean FV was 0.32 (SE, 96/200) ± 0.30 logMAR. Visual improvements were statistically significant (*p* < 0.001, paired *t*-test). The mean preoperative CFT was 466.71 ± 89.0 µm, and the mean final CFT was 353.75 ± 63.24 µm. The thickness resolution from before operation to final CFT was also significant (*p* < 0.001, paired *t*-test). ERM did not recur in any cases. The following conditions were noted postoperatively: pseudophakia (68.24%, 58/85), final EZ distortion (2.35%, 2/85), final central bouquet (11.76%, 10/85), and final foveal pit contour (28.24%, 24/85). The mean follow-up duration was 8.44 ± 7.28 months (3–34 months). Table 1 summarizes the correlation between preoperative and postoperative factors, such as preoperative and final visual and anatomical outcomes.

### 3.2. Functional Improvement

#### 3.2.1. Factors That Affected Final Vision

Final vision were significantly correlated with preoperative lens status (*p* = 0.026), preoperative vision [*p* = 0.005, correlation coefficient (CC): 0.3], preoperative IS/OS distortion (*p* = 0.029), final lens status (*p* = 0.001), and follow-up duration (*p* = 0.024, CC: −0.244). Stepwise multiple linear regression identified preoperative vision (*β* = 0.327, *p* = 0.010) and final lens status (*β* = 0.400, *p* = 0.002) as the only independent factors related to final vision (*R*^2^ = 0.309). Anatomical biomarkers, including EZ integrity and retinal thickness, provided no additional predictive value after controlling for these factors. By identifying both factors as independent predictors, the model disentangles final lens status from retinal recovery. The significance of preoperative vision (*p* = 0.010) emphasizes that baseline macular health remains the primary related factor, even when the confounding influence of postoperative lens status (*p* = 0.002) is mathematically controlled.

#### 3.2.2. Vision Improvement (Figure 2)

All patients showed a significant improvement (*p* < 0.001, paired *t*-test) from preoperative vision (0.47 ± 0.29 logMAR) to final vision (0.32 ± 0.30 logMAR). The mean vision score remained relatively stable one month postoperatively [0.47 (SE, 68/200) ± 0.33 logMAR], slightly improved at three months [0.41 (SE, 78/200) ± 0.35 logMAR], mildly declined at six months [0.47 (SE, 68/200) ± 0.40 logMAR], showed a slight improvement at twelve months [0.42 (SE, 76/200) ± 0.47 logMAR], and reached 0.32 (SE, 96/200) ± 0.30 logMAR at the last visit. In contrast, patients with pseudophakia experienced improvement from a mean preoperative vision score of 0.49 (SE, 64/200) ± 0.33 logMAR to 0.44 (SE, 72/200) ± 0.34 logMAR. By three months, it significantly improved to 0.27(SE, 108/200) ± 0.26 logMAR (*p* < 0.001) and remained steady at six and twelve months [0.29 (SE, 102/200) ± 0.26 logMAR] (*p* = 0.034). At the final follow-up, it slightly improved to 0.24 (SE, 116/200) ± 0.20 logMAR (*p* < 0.001). However, patients with phakia did not exhibit significant vision improvement throughout the study, except for those who underwent cataract surgery after the initial procedure. Their final vision showed significant improvement (*p* = 0.002, paired *t*-test), with a mean final vision score of 0.35 (SE, 90/200) ± 0.21 logMAR.

**Figure 2:**
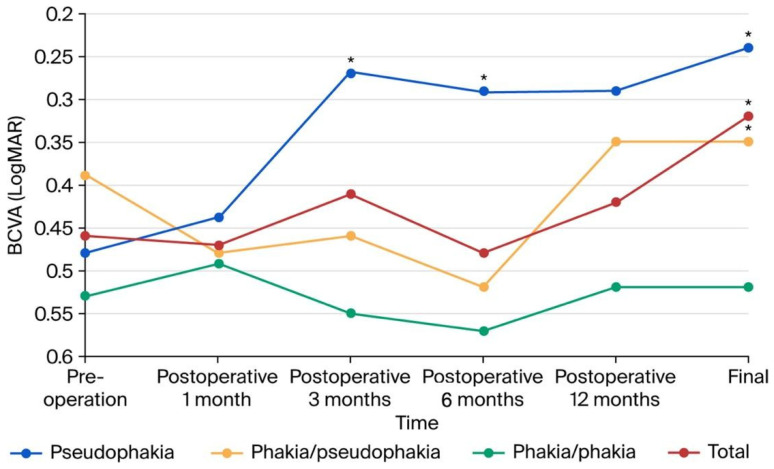
Vision changes among all patients and stratified groups according to preoperative and postoperative lens statuses. Patients with pseudophakia experienced a mild improvement in vision, with a mean preoperative vision score of 0.49 logMAR improving to 0.44 logMAR at one month postoperatively, significantly improving to 0.27 logMAR at three months postoperatively, and maintaining a steady level at 0.29 logMAR at six and twelve months postoperatively. The final vision scores slightly improved to 0.24 logMAR. However, there were significant differences in mean vision among the three groups at three and six months postoperatively and at the final visit. Regarding the final vision, patients with pseudophakia achieved the best outcome (0.24 logMAR), followed by those who initially had phakia and later underwent cataract surgery (0.35 logMAR), while patients who remained phakic throughout the study had the poorest final vision (0.52 logMAR). (*, *p* < 0.05; paired *t*-test).

### 3.3. Anatomical Improvement

#### 3.3.1. Factors Affecting the Final CFT

ERM stage (*p* = 0.006), preoperative OCT characters of intraretinal schisis-like cysts (*p* = 0.008) and central bouquet (*p* = 0.032), preoperative CFT (*p <* 0.001, CC: 0.558), preoperative IRLT (*p* = 0.002, CC: 0.330), preoperative ORLT (*p* < 0.001, CC: 0.503), final foveal pit contour (*p* < 0.001), follow-up duration (*p* = 0.006, CC: −0.296), final IRLT (*p* < 0.001, CC: 0.676), and final ORLT (*p* < 0.001, CC: 0.82) were significantly correlated with the final CFT. Stepwise regression for final CFT demonstrated high predictive value (*R*^2^ = 0.786, *p* < 0.001), identifying ERM stage (*β* = −0.383), preoperative CFT (*β* = 0.736), final IRLT (*β* = 0.652), final ORLT (*β* = 0.817), and final foveal pit contour (*β* = −0.212) as related factors influencing final foveal thickness. These results indicate that postoperative foveal thickness is determined by the residual integrity of both retinal layers and preoperative membrane severity.

#### 3.3.2. CFT Changes According to Different Lens Statuses (Figure 3A)

The CFT significantly decreased in thickness at one, three, six, and twelve months postoperatively, as well as the final visit (*p* < 0001, paired *t*-test). The CFT reduced from 466.71 ± 88.47 µm preoperatively to 392.29 ± 74.55 µm, 359.13 ± 63.85 µm, and 325.35 ± 58.3 µm at one, three, and six months postoperatively, respectively, maintaining a steady decrease at twelve months (338.29 ± 88.47 µm). Patients with preoperative pseudophakia showed consistently decreased thickness at one, three, and six months postoperatively (*p* < 0.001, paired *t*-test), at twelve months (*p* = 0.026), and at the final visit (*p* < 0.001). Patients with preoperative phakia throughout the study showed decreasing thickness at one, three (*p* < 0.001, paired *t*-test), and six months (*p* = 0.001), and at the final visit (*p* < 0.001). Meanwhile, patients initially phakic but ending as pseudophakic exhibited decreasing thickness at one, three, and six months (*p* < 0.001, paired *t*-test), at twelve months (*p* = 0.002), and at the final visit (*p* < 0.001).

#### 3.3.3. Inner Retinal Layer Thickness (IRLT) Changes According to Different Lens Statuses (Figure 3B)

There was a significant decrease in the IRLT observed at one, three, and six months postoperatively and at the final visit (*p* < 0.001, paired *t*-test). The IRLT reduced from a preoperative thickness of 125.60 ± 42.50 µm to 106.27 ± 37.41 µm one month postoperatively, 90.47 ± 34.79 µm at three months, and 75.45 ± 32.90 µm at six months, with a slight increase to 88.16 ± 36.68 µm at twelve months. Preoperatively pseudophakic eyes showed a noteworthy decrease in the IRLT at one (*p* = 0.038, paired *t*-test), three (*p* < 0.001), six (*p* = 0.026), and twelve months (*p* = 0.029), and at the final visit (*p* = 0.001). Patients with phakia throughout the study showed decreasing thickness at one (*p* = 0.005, paired *t*-test), and three months (*p* = 0.001), and at the final visit (*p* < 0.001). Conversely, patients initially phakic but becoming pseudophakic demonstrated a significant decrease in the IRLT at three (*p* = 0.022, paired *t*-test) and six months (*p* = 0.016), and at the final visit (*p* = 0.027).

#### 3.3.4. Outer Retinal Layer Thickness (ORLT) Changes in Different Lens Statuses (Figure 3C)

The ORLT significantly decreased at one, three, and six months postoperatively, as well as at the final visit (*p* < 0001, paired *t*-test). The ORLT decreased from a preoperative thickness of 345.78 ± 71.70 µm to 288.29 ± 50.51 µm at one month postoperatively, 266.35 ± 40.17 µm at three months, 251.61 ± 41.26 µm at six months, and 249.29 ± 48.20 µm at twelve months postoperatively. However, preoperative pseudophakic eyes showed a significant decrease in the ORLT at all postoperative time points (one and three months: *p* < 0.001, paired *t*-test; six months: *p* = 0.001; twelve months: *p* = 0.014; final visit: *p* < 0001). Patients maintaining phakia throughout the study displayed a significant reduction in the ORLT at one- and three-month time points (*p* < 0.001, paired *t*-test), six months (*p* = 0.010), and at the final follow-up (*p* < 0.001). In contrast, patients who were initially phakic but became pseudophakic also experienced a significant decline in the ORLT at one (*p* < 0.001, paired *t*-test), three, and six months, and at the final visit (*p* < 0.001).

**Figure 3:**
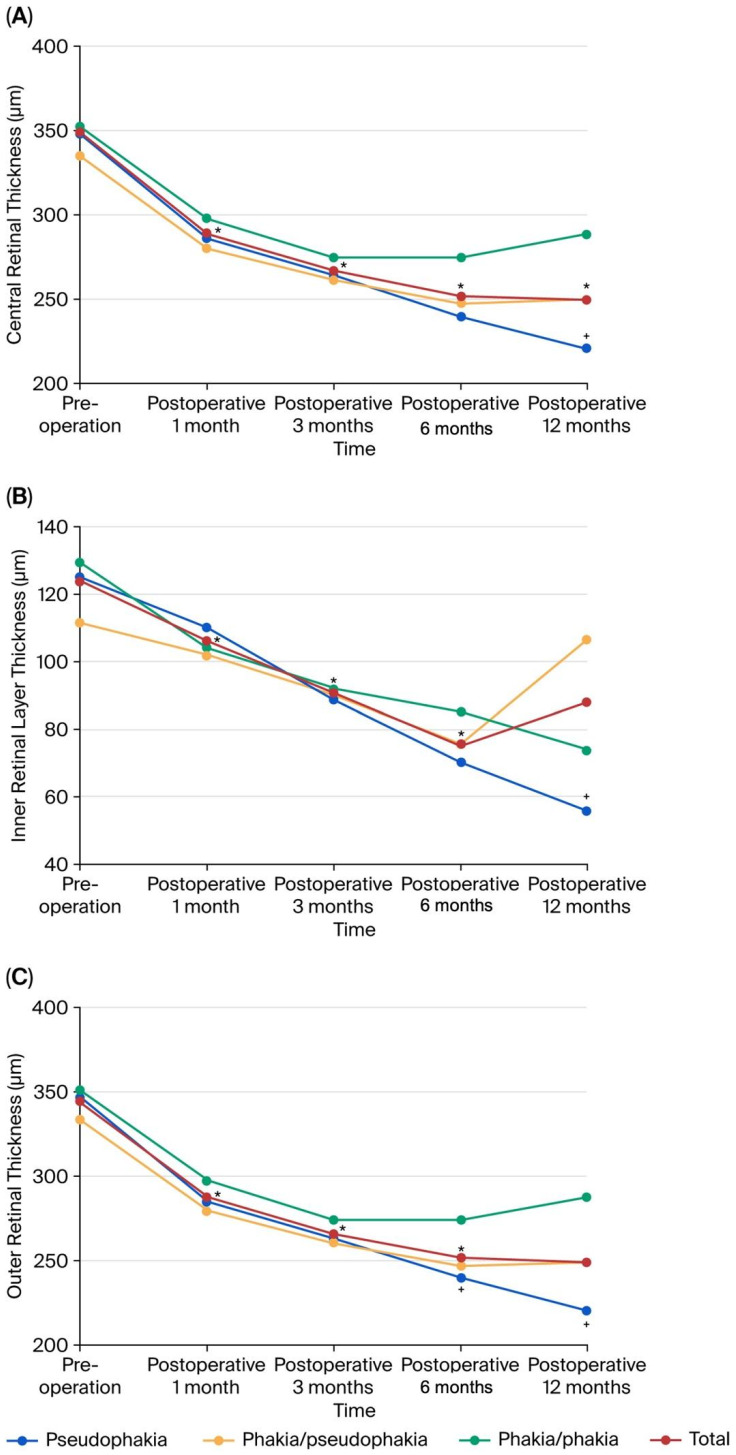
(**A**) The CFT changes in all patients according to different preoperative and postoperative lens statuses. The CFT of all patients decreased up to six months and remained stable up to twelve months postoperatively. Pseudophakic patients experienced a continuous CFT reduction up to twelve months postoperatively. Phakic patients throughout the study had a decrease in CFT up to three and six months postoperatively and then stability up to twelve months postoperatively. However, for initially phakic eyes that later underwent cataract surgery, the CFT decreased until six months postoperatively and then showed a gradual increase up to twelve months postoperatively. (*, *p* < 0.05, paired *t*-test; +, *p* < 0.05, independent *t*-test, pseudophakia group vs. phakia group). (**B**) The IRLT changes in all patients and in patients with different preoperative and postoperative lens statuses. All patients experienced a decrease up to six months postoperatively, followed by an increase up to twelve months postoperatively. Patients with pseudophakia showed a continuous decrease in the IRLT up to twelve months postoperatively. In the group of patients with phakia throughout the study, the IRLT decreased gradually up to twelve months postoperatively. Similarly, for initially phakic eyes that underwent cataract surgery later, the IRLT decreased until six months postoperatively and then increased notably up to twelve months postoperatively. (*, *p* < 0.05, paired *t*-test; +, *p* < 0.05, independent *t*-test, pseudophakia group vs. initially phakic but later pseudophakic group). (**C**) The ORLT changes in all patients according to different preoperative and postoperative lens statuses. The ORLT in all patients decreased up to six months postoperatively and then remained stable up to twelve months postoperatively. Patients with pseudophakia experienced a continuous decrease in ORLT up to twelve months postoperatively. In the group of patients with phakia throughout the study, the ORLT decreased until three months postoperatively, remained stable until six months, and then gradually increased up to twelve months postoperatively. For initially phakic eyes that underwent cataract surgery later, the ORLT decreased until six months postoperatively and then remained stable up to twelve months postoperatively (*, *p* < 0.05, paired *t*-test; +, *p* < 0.05, independent *t*-test, pseudophakia group vs. phakic throughout the study group).

## 4. Discussion

Preoperative OCT-based biomarkers of iERM that were predicted to affect the final visual prognosis have been reported in previous studies, including IRF [16], changes or irregularities of the inner retinal layers [8,17,18], disorganization of the retinal inner layer (DRIL) [19], central bouquet [15,20], photoreceptor or EZ integrity [12,13,21,22,23,24], and the presence of subfoveal detachment [25]. Lee et al. [16] found that the preoperative presence of microcystic macular edema might be a prognostic factor for poor visual recovery after surgery. However, in our study, there was no significant difference in final vision and CFT regardless of whether intraretinal cysts were present; however, schisis-like cysts were correlated with a larger final CFT but not with final visual outcome, which is consistent with previous studies [26,27,28]. As for central bouquet abnormalities and tractional abnormalities over the central foveal cones, this specific structure usually resolves with vision improvement after surgery, but the visual prognosis varies in cases with or without it. While previous studies [15,20,29] reported similar univariate associations, our multivariate stepwise regression further clarifies their role by identifying which factors remain independent predictors of final visual outcome. According to iERM grading, the presence of EIFLs and disorganized retinal layers indicated severe iERM with poor preoperative vision compatible with Yang et al.’s study [18]. They proposed that preoperative vision in cases with EIFL showed significant differences from those without EIFL in the early postoperative period (<4 months), but no difference was observed 10 months postoperatively. Additionally, our study found no final vision difference in iERM stages I/II and III/IV at a mean of 8.44 months.

Furthermore, Zur et al. [19] found better preoperative vision in patients with and without mild DRIL than in those with severe DRIL. They concluded that DRIL could serve as a biomarker for predicting postoperative outcomes. However, we found that the final vision of patients with DRIL (iERM stages I/II/III) was not significantly different from that of patients without DRIL (iERM stage IV), which may be because few patients in our study had DRIL or severe DRIL. Regarding the integrity of photoreceptors or the EZ, Mitamura et al. [12] reported that the recovery of EZ defects may result in better visual outcomes related to the morphological and functional recovery of photoreceptor cells. Conversely, Suh et al. [13] reported that photoreceptor disruption due to macular traction by ERM seldom recovers and results in poor visual outcomes after surgery. They reported that only 22.7% and 31.8% of eyes with preoperative EZ disruption were reversed three and six months after surgery, respectively. Despite univariate significance, EZ distortion was not an independent factor in our multivariate analysis, suggesting that photoreceptor damage is largely reflected in preoperative vision. However, the anatomical recovery rate of the EZ was notably high at 85.71% (12/14). This indicates that while structural restoration of the EZ is a common postoperative feature, preoperative functional status remains the primary determinant of visual success in our cohort. Another factor related to final vision in our study was final lens status. As shown in Figure 2, the postoperative vision in the pseudophakia group achieved the best mean vision from one month to the endpoint, while the group with initially phakic eyes that became pseudophakic postoperatively achieved better mean vision than the group with phakia throughout the study. By adjusting for lens status, we confirmed that preoperative retinal function is the primary determinant of postoperative success. The high predictive value of our structural model (*R*^2^ = 0.786) further validates that anatomical recovery occurs independently of lens-related confounding.

Bae et al. [30] reported that patients with foveal pit restoration after surgery showed no significant difference in BCVA but showed a thinner CFT than the non-restoration group at the one-year follow-up. Suh et al. [13] reported that eyes that recovered to a normal concave foveal shape did not present better visual acuity than other types, indicating that the inner retinal structure might not be the main determining factor of postoperative visual outcomes. Similar results were obtained in our study, in which the recovery rate of the foveal pit contour was 28.24%, which was not significantly different from the final vision scores. Following the recovery of distension or traction of intraretinal cells and fibers without EZ, the final vision improved faster than the CFT, which may gradually return to normal thickness and contour. Regarding anatomical outcomes, including EILF, DRIL, intraretinal schisis-like cysts, central bouquets, preoperative CFT, preoperative IRLT, preoperative ORLT, and final foveal pit contour, factors related to the CFT seemed to affect temporary vision but not final vision. Regarding anatomical recovery, we found that the CFT decreased continuously until twelve months postoperatively in the pseudophakia group, while the CFT in the phakia group decreased in the first three months postoperatively but was maintained or mildly increased in the last nine months. The CFT in the group of patients with preoperative phakia and a final outcome of pseudophakia decreased in the first six months but slowed down thereafter.

We also analyzed both the inner and outer retinal layers to assess anatomical recovery in different lens statuses. In the pseudophakia group, both the IRLT and ORLT continuously decreased postoperatively. In the phakia group, both the IRLT and ORLT decreased until six months postoperatively and remained steady thereafter. In the group with preoperative phakia but a final outcome of pseudophakia, the IRLT decreased within six months postoperatively but increased later, whereas the ORLT decreased within six months but remained steady later. This study is limited by its retrospective design and a variable follow-up period (3–34 months). However, multivariate stepwise regression demonstrated that follow-up duration was not an independent factor of either functional (*p* = 0.888) or anatomical (*p* = 0.241) outcomes, suggesting that the observed retinal recovery had reached a stable plateau in our cohort.

## 5. Conclusions

The factors influencing final vision included preoperative vision and final lens status. Temporary visual changes and long-term anatomical recovery were influenced by factors such as ERM stage, preoperative CFT and ORLT, final foveal pit contour, final IRLT and ORLT. In pseudophakic patients, vision significantly improved to a plateau of 102/200-108/200 within three months, alongside continuous anatomical thinning over twelve months. Conversely, patients with phakic eyes exhibited the poorest functional outcome (60/200), with continuous anatomical thinning in the first 6 months and then stability. Furthermore, patients who were initially phakic but later had pseudophakia achieved superior final vision (90/200) compared to patients whose eyes remained phakic, despite a unique secondary increase in the IRLT during the late postoperative period.

## Figures and Tables

**Table 1:** Correlation of preoperative and postoperative factors with preoperative and final visual and anatomical outcomes.

Total Eyes: 85 Total Patients: 85	n (%) Mean (SD)	*p* (Final Vision)	*p* (Final CFT)
**Age,** years, mean (SD)	65.64 (6.19)	0.100 ^a^	0.649 ^a^
**Male,** n (%)	30 (46.15)	0.777 ^b^	0.754 ^b^
**Eye (OD),** n (%)	47 (55.29)	0.146 ^b^	0.114 ^b^
**ERM OU,** n (%)	20 (24.0)	0.345 ^b^	0.675 ^b^
**Axial length,** mm, mean (SD)	23.96 (1.00)	0.474 ^a^	0.171 ^a^
**Preoperative factors**			
**Lens status (pseudophakia),** n (%)	36 (42.35)	0.026 ^a^*	0.877 ^a^
**Pre-op vision,** logMAR, mean (SD)	0.47 (0.29)	0.005 ^a^*	0.165 ^a^
**Stage,** n (%)		0.361 ^b^	0.006 ^b^*
**I/II**	18 (21.18)		
**III/IV**	67 (78.82)		
**I/II/III** **IV**	69 (81.17) 15 (17.65)	0.491 ^b^	0.042 ^b^*
**OCT characters,** n (%)			
**Intraretinal cyst**	68 (80.00)	0.961 ^b^	0.500 ^b^
**Schisis-like cyst**	16 (18.82)	0.682 ^b^	0.008 ^b^*
**EZ distortion**	14 (16.47)	0.029 ^b^*	0.580 ^b^
**Central bouquet**	35 (41.18)	0.429 ^b^	0.032 ^b^*
**Pre-op CFT,** µm, mean (SD)	464.20 (89.00)	0.786 ^a^	<0.001 ^a^*
**Pre-op IRLT,** µm, mean (SD)	122.04 (41.92)	0.866 ^a^	0.002 ^a^*
**Pre-op ORLT,** µm, mean (SD)	341.73 (71.54)	0.692 ^a^	<0.001 ^a^*
**Postoperative factors**			
**Final lens status (pseudophakia),** n (%)	58 (68.24)	0.001 ^b^*	0.251 ^b^
**Final vision,** log MAR, mean (SD)	0.32 (0.30)		0.289 ^a^
**Final EZ distortion,** n (%)	2 (2.35)	0.555 ^b^	0.534 ^b^
**Final central bouquet,** n (%)	10 (11.76)	0.938 ^b^	0.777 ^b^
**Final foveal pit contour,** n (%)	24 (28.24)	0.597 ^b^	<0.001 ^b^*
**Follow-up,** month, mean (SD)	8.44 (7.28)	0.024 ^a^*	0.006 ^a^*
**Final CFT,** µm, mean (SD)	351.33 (63.24)	0.289 ^a^	
**Final IRLT,** µm, mean (SD)	88.05 (36.32)	0.332 ^a^	<0.001 ^a^*
**Final ORLT,** µm, mean (SD)	265.62 (46.82)	0.063	<0.001 ^a^*

* *p* < 0.05, ^a^ Pearson correlation, ^b^ independent *t*-test; n: number; %: percentage; SD: standard deviation; ERM: epiretinal membrane; OU: oculus uterque; Pre-op: preoperative; logMAR: logarithm of the minimum angle of resolution; OCT: optical coherence tomography; EZ: ellipsoid zone; CFT: central foveal thickness; IRLT: inner retinal layer thickness; ORLT: outer retinal layer thickness.

## Data Availability

All data supporting our study will be shared upon request, although the majority is contained within the manuscript.

## Abbreviations

iERM: idiopathic epiretinal membranes; ILM: internal limiting membrane; OCT: optic coherence tomography; SE: Snellen equivalent; EZ: ellipsoid zone; PVD: posterior vitreous detachment; PPV: plana vitrectomy; CFT: central foveal thickness; BCVA: best-corrected visual acuity; logMAR: the logarithm of the minimum angle of resolution; RLT: retinal layer thickness; IRLT: inner retinal layer thickness; ORLT: outer retinal layer thickness; EIFLs: ectopic inner foveal layers; INL: inner nuclear layer; IPL: inner plexiform layer; IRL: inner retinal layer; ORL: outer retinal layer; CC: correlation coefficient; DRIL: disorganization of the retinal inner layer
